# School Achievement and Performance in Chilean High Schools: The Mediating Role of Subjective Wellbeing in School-Related Evaluations

**DOI:** 10.3389/fpsyg.2017.01189

**Published:** 2017-07-14

**Authors:** Verónica López, Juan C. Oyanedel, Marian Bilbao, Javier Torres, Denise Oyarzún, Macarena Morales, Paula Ascorra, Claudia Carrasco

**Affiliations:** ^1^School of Psychology, Pontificia Universidad Católica de Valparaíso Valparaíso, Chile; ^2^Centro de Investigación para la Educación Inclusiva Valparaíso, Chile; ^3^Faculty of Education, Universidad Andrés Bello Santiago, Chile; ^4^School of Psychology, Universidad de Santiago de Chile Santiago, Chile; ^5^Universidad Tecnológica de Chile INACAP Vitacura, Chile

**Keywords:** school achievement, school failure, student wellbeing, school climate, social wellbeing, Latin America, high school, Chile

## Abstract

School achievement gaps and school failure are problematic issues in Latin America, and are mainly explained by the socio-economic status (SES) of the students. What schools can do to improve school achievement and reduce school failure is a critical issue, both for school management and teacher training. In this study, we present the association of individual and school-related socio-emotional variables with school achievement and performance, controlling for the effects of SES. A probabilistic sample of 4,964 students, drawn from 191 schools enrolled in year 10 in urban areas of Chile, answered questionnaires assessing subjective wellbeing, social wellbeing in school, school climate, school social wellbeing and students’ perceptions of teachers’ wellbeing. Using structural equation modeling, and controlling for SES, we modeled subjective wellbeing as a mediator of the relationship between school-related variables, such as school climate and perception of teacher’s wellbeing, and (a) school achievement, and (b) school performance. School achievement was computed as a product of (a) the probability of passing the school year, and (b) the percentage of yearly attendance at school. Data on school achievement was drawn from administrative registries from the Chilean Ministry of Education. School performance was computed as the estimated grade point average (GPA) at the end of the school year, based on the students’ previous 5-year GPAs, and was also obtained through administrative data of the last 5 years. Findings reveal the mediating role of subjective wellbeing in the relationship between school-related evaluations (students’ social wellbeing at school, their perception of teachers’ wellbeing and school climate) and school achievement. For school achievement, two variables were mediated (students’ social wellbeing at school and school climate). However, for school performance, no significant mediations were found. We conclude that, on the one hand, after controlling for SES, students’ individual subjective wellbeing is associated with their achievement and performance in school. We discuss the importance of improving school experiences that may protect and promote students’ subjective experience and school achievement and performance, and reduce the probability of school failure and dropout.

## Introduction

School achievement gaps and school failure are problematic issues in Latin America. Although enrolment has increased and general dropout has decreased, graduation rates are low, and these indicators show important gaps regarding gender, regions within countries and socio-economic groups (Bassi et al., 2013).

In Chile, dropout rates are lower than in other Latin American countries reaching 5.4% in 2013^1^. However, this percentage hides a socio-economic gap, with 32.6% of students who drop out being from the lowest socio-economic status (SES) (CASEN, 2013). Research suggests that Chile has one of the most socio-economically segregated educational systems in the world (OECD, 2011; Valenzuela et al., 2014), raising the issue of what schools can do to improve school achievement and reduce school failure.

School dropout has consequences both for those leaving the school system and for society at large (Rumberger and Rotermund, 2012). Dropouts are less likely to find employment and are more likely to end up earning lower wages. There is also evidence that school dropout is a predictor of delinquency, violence and drug use during adolescence and early adulthood (Henry et al., 2012; Na, 2016).

Research has addressed causes of school dropout, highlighting among them violent behavior and unhealthy peer relationships (Finn and Rock, 1997), and low parental commitment to the education of their sons and daughters (Pölkki and Vornanen, 2015). Also, it has noted the role of low attachment to school and low subjective and psychological wellbeing (South et al., 2007).

Other factors highlighted by the literature include the SES of the family, and social vulnerability. There is usually marked by an early incorporation into labor, adolescent pregnancy, poor academic performance, lack of motivation to study, behavioral problems, high school and neighborhood mobility, and grade repetition (Cairns et al., 1989; Aguirre et al., 2009; Román, 2009; Tyler and Lofstrom, 2009; Gasper et al., 2012. In the case of Chile, see Herrera, 1999; García-Huidobro, 2000; CEPAL, 2002; Román, 2009; Espinoza et al., 2011; Espinoza et al., 2012; Espinoza-Díaz et al., 2014).

Grade repetition and school non-attendance have been pinpointed as specific and measurable indicators of estimated school dropout (Havik et al., 2015). Grade repetition, as well as school dropout, generates what is known as educational lag, which is defined as a gap between the educational level a student has at a given age and the educational level which he or she is supposed to have according to the national normative standards.

School-related evaluations could have a strong impact on student achievement and behavior (school achievement). Nonetheless, it is important to consider the individual evaluation that adolescents do of their own lives and how it affects both their psychological, behavioral and educational outcomes. To assess their own appraisal of their lives it is worth to look at the literature of subjective wellbeing.

Subjective wellbeing is understood as “an umbrella term for different valuations that people make regarding their lives, the events happening to them, their bodies and minds, and the circumstances in which they live” (Diener, 2006, p. 400). Subjective wellbeing has proven to be critical to maintaining positive mental health. Among its determinants are also the social aspects that mark people’s lives (Keyes and Lopez, 2005).

Current research on happiness and subjective wellbeing is characterized by three main perspectives: subjective wellbeing (Diener, 2006), psychological wellbeing (Ryff and Singer, 1998), and social wellbeing (Keyes, 1998, 2006; Keyes, 2013). All these perspectives offer an interesting dimension: the relationship of wellbeing with the concept of health, which has as its protagonist an active and socio-historical subject (Blanco and Valera, 2007).

Subjective wellbeing studies, according to the guidelines of OECD (2013), include three major aspects: affectivity (positive, negative, and balance of affects), overall life assessment (life satisfaction and life domains), and sense of life (perception of living a life with meaning and purpose, and good psychological and social functioning). The first two aspects have traditionally been developed by a hedonic perspective such as the studies of happiness, while aspects of the psychological functioning of life with meaning and purpose come from studies on the tradition of eudemonics (Vázquez and Hervás, 2008; OECD, 2013).

Keyes proposes the relevance of the evaluation of our social functioning, and our evaluative perception of society in general, as one of the pillars of positive mental health (Keyes, 1998; Keyes and Shapiro, 2004; Keyes and Lopez, 2005). Social wellbeing is a complementary pillar to psychological wellbeing, which contributes to the construction of life with meaning and purpose by enabling meaningful relationships with others. This leads to a feeling of relevance in the social world, which is intelligible, and has a history and future to which the person feels attached (Keyes, 2006).

Social wellbeing has been defined as “the evaluation we make of circumstances and functioning within society” (Keyes, 1998, p. 7). Social integration, social acceptance, social contribution, social updating, and social coherence are the key areas defining social wellbeing (Keyes, 1998; Keyes and Shapiro, 2004). Thus, social wellbeing evaluates the interpersonal aspects of mental health. Considering these dimensions, the measurement of social wellbeing has become increasingly important in recent years, mainly due to its relations with civic health and social capital (Putnam, 2001) and especially with mental health from a biopsychosocial perspective (Keyes and Shapiro, 2004; Keyes, 2013).

Studies also show that environmental circumstances can sometimes produce substantial and lasting differences in subjective wellbeing (Diener, 2006; Oyanedel et al., 2014). One of these areas is school experience. The school can be seen as a place where subjective wellbeing becomes greatly important in the formation of adolescents. It is at this stage that an important part of their future satisfaction with life is defined (Cárdenas et al., 2008), as well as the definition of their future projects, and their relational and self-perception frameworks. Thus, high school constitutes an important stage in the development of students’ cognitive, social and emotional capacities. It is precisely at this stage of life that adolescents construct an image of themselves, get in touch with their peers and begin to experience and control a wide range of emotions (Ning et al., 2013).

Most research on adolescent life satisfaction has examined the roles of family functioning and intrapersonal variables, but few studies have investigated life satisfaction in relation to schooling (Suldo et al., 2006; Wit et al., 2011; Veltro et al., 2014). Existing research linking satisfaction with life and satisfaction with the school shows that the most relevant associations are found with achieving a good school performance (Huebner and Gilman, 2006; Dwyer, 2008; Kirkcaldy et al., 2009; Saab and Klinger, 2010; Diseth et al., 2012), with a good perception of the quality of education received (Cárdenas et al., 2008), with an increase in the perception of social opportunities through education (Ferrante, 2002), with the perceived support of the social context in which young people develop (Hirschi, 2009) and finally, with the objective measurement of school performance (Quinn and Duckworth, 2007).

Students’ life satisfaction is negatively affected by poor school climate and instances of school violence. Research has shown significant relationships between low levels of satisfaction with life and greater violence (physical and psychological) received from the peer group members (Oyanedel and Bazan, 2011), and where aggressive behavior is used as a social recognition mechanism (Buelga et al., 2008).

This research advances the understanding of the subjective wellbeing of adolescents, as it also considers social wellbeing (Keyes, 1998) within the school world, from a social-ecological perspective (Bronfenbrenner, 1987; Bronfenbrenner and Morris, 2006; Espelage and Swearer, 2010). If schools can make a significant difference in the integral development of students by promoting subjective (psycho-socio-affective) variables related to their wellbeing, there may be an alternative to the structural determinism derived from the segregation (based on socio-economic level) of the Chilean educational system.

The promotion by teachers and managers of healthy school environments favors the integral development of students within the framework of a school culture that cares about the quality of life of their community (Benbenishty and Astor, 2005; López et al., 2011). A healthy school environment should affect academic achievement, understood not only through the probability of obtaining a better academic performance but also as the likelihood of preventing school failure and dropout.

Assessing the relationship between subjective wellbeing and educational achievement requires the development of more sophisticated models to avoid two possible restrictions. The first is the effect of SES, which, as reported by previous research, shows significant association both with the dependent and independent variables. The second is associated with focusing only on associations, instead of disentangling the mechanisms behind these associations. In this study, we propose that, at least, two supplementary school-related factors could be involved in this relationship: the role of pedagogical support of teachers, and school climate.

Recently, studies about educational quality have highlighted the role of the school context among its determinants. From a socio-ecological perspective (Benbenishty and Astor, 2005; Bronfenbrenner and Morris, 2006; Espelage and Swearer, 2010), student learning outcomes should consider the interrelation between the different levels that operate in and through the school system. From this perspective (Bronfenbrenner, 1987; Khoury-Kassabri et al., 2004), phenomena such as school violence are the result of the interaction between different relevant subsystems, among them students, families, the school, and the general community. All play a determinant role in individual performance.

School climate corresponds to a multidimensional construct related to the perceptions, thoughts, and values that members of an educational establishment give to it, and the social relations taking place on it (Assael and Neumann, 1991; Benbenishty and Astor, 2005). It is a characteristic of educational establishments, produced by the perceptions of the students about certain variables which, in turn, generate perceptions about that school. It has a strong impact on students’ behavior and academic results. School climate is a phenomenon different from bullying and school violence (Astor et al., 2006).

School climate appears to have a strong effect on both educational attainment and the development of psychological strenghts. Literature on school climate makes clear that, although broad, it is generally understood under four specific dimensions: academic, community, safety and, institutional (Wang and Degol, 2016). Even when in practice they overlap, each of these represents an specific area of analysis and intervention in school settings.

The academic dimension of school climate, is one of the most prominent areas of school climate, dealing mainly with the importance of teaching and learning activities inside the school, and one of the most widely studied (Thapa et al., 2013). The community level, referring to the role that relationships have inside the school has been also researched widely, both in terms of academic outcomes, as well as their role in promoting positive psychosocial adjustment, increasing connectedness and reducing disengagement. School engagement has been reported as a protective factor for school dropout and school failure (Fall and Roberts, 2012; Wang and Fredricks, 2014). Engagement also shows association with intrinsic motivation and high educational expectations (Fan and Wolters, 2012), as well as preventing depression and low self-efficacy in academic settings (Quiroga et al., 2013). Benbenishty and Astor (2005) have specified three elements involved in generating a favorable school climate: clear policies and standards (Johnson, 2010); positive and supportive relationships with adults (Dwyer et al., 2001), and subjective and social wellbeing of students, insofar as positive relationships favor trust and commitment to the school.

Creating a healthy school climate is then a key role of the management team and teachers. They are in charge of building a supportive environment for learning, not only from individual attitudes of respect for diversity, solidarity and good treatment but also by establishing management practices which make it possible to build and sustain these relationships over time (Wubbels, 2011; Wubbels et al., 2015). Managing school behavior, promoting a feeling of belonging to the school, and establishing a system of legitimate and fair rules requires an active management of coexistence. It also requires a democratic style of management, where the participation of the school community is valued and practices allowing this participation are generated and promoted (López et al., 2011).

Comparative research shows the relationship between school failure and negative school climate (McEvoy and Welker, 2000). In the United States, Werblow et al. (2010) show that a positive school climate predicts school retention and, therefore, prevents dropout. The SERCE (UNESCO, 2008) and TERCE studies (UNESCO, 2016), carried among students of primary schools in Latin American countries, also found this relationship to apply with achievement in language, mathematics, and science. Scherer and Nilsen (2016) report, using a large-scale comparative dataset, an association between positive school climate and students’ academic achievement motivation in mathematics, although of lesser extend that the association between achievement and instructional quality.

Samdal et al. (1998) examined the relationship between school climate and school satisfaction using data for students aged 11, 13, and 15 in Finland, Latvia, Norway, and Slovakia. Among school climate factors (teacher support, student support, classroom disturbances, unreasonable job demands, school justice, school safety, harassment and loneliness during time between classes), the authors found that the strongest predictors of student satisfaction with school are organized into process factors (school justice and school safety) and teacher support. These are followed by student support and class disturbances.

Wentzel (1998), using a sample of 167 sixth graders in a suburban community in the United States, found that academic support from teachers and peers is related to interest in school. Natvig et al. (2003) explored the relationship between happiness and stress in school, as well as personal and social factors associated with them, among 887 Norwegian adolescents aged 10–15 years old. They found that teacher support and peer support were positively related to happiness and that the support of teachers seemed to be more important than the support of other students.

In summary, available research shows that academic achievement and school climate are associated with adolescent subjective wellbeing.

Those teachers who favor an environment in their classrooms centered on learning and who play a role as mediators of this learning process, tend to achieve better results in terms of student learning and school performance (Ascorra et al., 2003; Davis, 2003; Ascorra and Crespo, 2004; Quaas et al., 2005; Pianta et al., 2012). In Latin America, the second and third international comparative study on language, mathematics and associated factors developed by UNESCO (UNESCO, 2008, 2016) reported that students who obtain the best results come from classrooms where students do not bother each other, where there are few or no fights, and where most of them are friends. Both reports conclude that classroom climate affects the academic success of students, so it is a key factor to consider in interventions aimed at educational improvements.

Several authors have emphasized the relevance of classroom climate and school coexistence to facilitate a learning-centered environment (Fraser, 1996; Adelman and Taylor, 1997; Hamre and Pianta, 2006; López et al., 2012; Friedberg, 2015). Literature also focus on the student-teacher relationship, which can be understood as a dyadic system. This relationship involves both characteristics of the student as well as of the teacher (Prino et al., 2016). This relationship is embedded in the school culture, which can regulate and affect it. Recent research reports that the student-teacher relation can operate as a protective factor in transitional events, for instance in the integration of students to new school (Longobardi et al., 2016) as well as in the integration of students with special needs (Pasta et al., 2013).

Research on effective schools (Raczynski and Muñoz, 2005; Allen et al., 2013), emphasizes the role of teachers as key actors in the processes carried out in classrooms, highlighting the construction of a good coexistence and a classroom climate based on learning, and on the choice of cooperative methodologies. Understanding teachers as professionals capable of thinking and making decisions about the conditions under which learning is developed (López-Vargas and Basto-Torrado, 2010) opens a space for a better management of classroom climate. There may be a relationship between teachers’ behaviors and the development of a classroom climate capable of promoting learning. Furthermore, the existence of a positive classroom and school climate builds and maintains not only positive teacher-student relationships, but also a higher sense of wellbeing for teachers themselves (Allen et al., 2013). In this study, we propose that students can perceive their teachers’ wellbeing and that this perception influences their school achievement and performance.

This study aims to understand the relationship between high school students’ school social wellbeing, school climate, teachers’ perceived wellbeing and academic outcomes. We hypothesize positive associations between these constructs. We also hypothesize that subjective wellbeing could act as a mediator of some of these relationships, meaning that for high school students, subjective wellbeing plays an important role for the interpretation and understanding of school-related phenomena.

## Materials and Methods

### Participants and Procedure

This study uses a probabilistic, stratified and two-stage (school-classroom) sample of students in regular high schools in urban zones of the three main regions of Chile (V, VIII, and the Metropolitan region of Santiago de Chile). The sampling framework used was the 2012 national school enrolment registry from the Chilean Ministry of Education, and these data were linked with the one of the National School Vulnerability Index (IVE-SINAE) to include SES of the school. In the first stage, schools teaching secondary education were stratified, and a random selection made within each stratum. Then, classrooms of second degree of secondary school were selected using Kish table.

Our selected sample was composed of 221 educational establishments. Asking for institutional consent, we received a rejection rate of 13.6% resulting in a final sample of 191 establishments (**Table 1**).

**Table 1:** Sample distribution by socio-economic status, administrative dependency, and region.

Sample	Socio-economic status									
	High			Medium			Low			
	V	VIII	RM	V	VIII	RM	V	VIII	RM	Total
Private	13	11	13	0	0	0	0	0	0	37
Voucher	13	15	13	13	12	10	4	5	11	96
Public	0	1	7	6	11	6	8	12	7	58
Total	26	27	33	19	23	16	12	17	18	191

Fieldwork was carried out during August and September 2013. The estimated student sample was 5,367^2^, equating to a response rate of 92.4%. The resulting sample consisted of 4,964 students. The sample size is associated with an observed maximum error of ±1.4%, assuming a maximum variance and a 95% confidence level. At the regional level, the absolute error is ±2.4%.

In this study, 51% of participants were men. Most respondents attended schools in the VIII Region (36.2%), followed by the Metropolitan Region (35.9%), and finally by the V Region (27.8%). Most of the sample were voucher schools (54.1%) [(Junta Nacional de Auxilio Escolar y Becas (JUNAEB)], while 30.7% were public schools, and 15.1% were private schools. Most schools in the sample belonged to the high socio-economic group (46%), followed by middle-level (33.7%), and low (20.3%).

### Ethical Considerations

We followed a two-stage consent process: First, school principals gave their consent for the adolescents participating in our study. Individual informed consent to take part in the research was also collected from the adolescents, along with written consent describing the nature and objective of the study following the ethical code of the Chilean National Commission for Science and Technology. The consent stated that data confidentiality would be assured and participation was voluntary. For the adolescents, representatives of each school parents’ association were asked to sign a consent form to have their children participate in our study. An information document was sent to each student’s parents explaining the research and including a clause allowing them to exclude their child from it. The study was approved by the IRB of the Pontifical Catholic University of Valparaíso.

### Measures

Differencing school social wellbeing and students’ subjective wellbeing is an important element of this research. Whereas subjective wellbeing focuses on individual experience, school wellbeing looks at the work of a school as a system. Subjective well-being refers to a person’s cognitive and affective evaluations of his or her life, including both emotional reactions as well as cognitive judgments of satisfaction (Diener et al., 2002: 63). Similarly, child subjective wellbeing should be understood as an individual conviction of a young person about the degree of accomplishment of his/her living needs, approached regarding satisfaction, happiness, fears and apprehensions (Strózik et al., 2016). Subjective wellbeing is composed of both an emotional and cognitive dimension. While emotional wellbeing involves an excess of positive over negative feelings, personal psychological functioning involves the presence of more positive than negatively perceived self-attributes (Keyes, 1998: 122). On the other hand, School social wellbeing is an adaptation of the construct of social wellbeing (Keyes, 1998) to school settings. Social wellbeing highlights the role that social life and social roles play in the constitution of self and represents a more eudemonic measure of wellbeing. School social wellbeing is a measure of the social health of the school, as perceived by individual students. It is more linked to the “community level” in school climate research (Wang and Degol, 2016), in the way it relates to an appraisal of a system of relationships taking place in the school, considering dimensions associated with integration, acceptance, and contribution.

The following measures were used:

#### Subjective Wellbeing

##### Personal wellbeing index (Casas and Bello, 2012)

The Personal wellbeing index (PWI) was designed by Cummins et al. (2003). It initially included seven items related to satisfaction with different areas of life: health, the standard of living, achievements, safety, belonging groups, future security, and interpersonal relationships. It later incorporated school children’s satisfaction with their situation, and with school life. This nine-item Likert scale, with a response range of 0 (“totally unsatisfied”) to 10 (“fully satisfied”), has shown a good performance in adolescents (12 years old and older). The results of this scale in Chile were satisfactory, with an alpha for the total scale of 0.83, forming a single factor that explains 44.5% of the variance (Bilbao et al., 2016). In our sample, factor analysis confirms the configuration of a single factor, with α = 0.81 for the nine items. For the construction of this index, the raw scores of the items were added.

##### Brief multidimensional students’ life satisfaction scale – BMSLSS – (Seligson et al., 2003)

This brief scale assesses six areas of satisfaction: family life, friends, school experience, the students themselves, the place where the student live, and overall satisfaction with life. The responses ranges of items range from 1 (“very unsatisfied”) to 7 (“very satisfied”). The scale showed a single factor with a reliability of α = 0.80. Raw scores were added to create the additive scale.

##### Positive and negative affect schedule (Watson et al., 1988)

This scale consists of 20 items measuring the occurrence of specific kinds of affect over the previous month. It is divided into two dimensions of ten items each, focusing on positive emotions (e.g., attentive, interested, proud), and on negative emotions (e.g., fearful, irritable, concerned). Items are on a 5-category Likert scale (where: 1 = “nothing or very slightly” and 5 = “extremely”). The reliability was α = 0.80 for the total scale, with a factorial structure for Negative (α = 0.81) and Positive Affectivity (α = 0.83). For the construction of these indices, the raw scores of the items were added and then averaged. After this, the “Balance of Affects” index was constructed, by subtracting the sum of the items showing Negative Affectivity from those showing Positive Affectivity.

#### School Social Wellbeing

To measure social wellbeing in school settings, we adapted Keyes’ Social Wellbeing Scale (1998). The original scale seeks to know individuals’ perceptions of the functioning of society, and their role in it. To estimate the level of wellbeing placed in a student context, the word “society” was changed to “school” and the language culturally adapted following expert advice and previous research experience. The scale showed a good psychometric behavior in school settings, with an α for the total scale of 0.88, and concurrent validity with other instruments that evaluated complementary constructs such as classroom climate and school climate. Confirmatory factor analysis with primary school students presented an adequate structure with three dimensions: “Social Integration,” “Social Acceptance,” and “Social Contribution” (CFI = 0.93; RMSEA = 0.046). The adaptation for adults, presented an adequate structure of the five dimensions, adding “Social cohesion” and “Social updating” (CFI = 0.91; RMSEA = 0.075).

#### School Climate

The school climate scale [Benbenishty and Astor, 2005, adapted and validated by López et al. (2014)], measures three dimensions: “norms,” “participation,” and “teachers’ support,” showing good behavior in different school populations (α = 0.87). Items range from 1 (“strongly disagree”) to 5 (“strongly agree”). Confirmatory factor analysis showed a three-dimensional structure (CFI = 0.946; RMSEA = 0.049) indicating a good fit of the model to the general population. For the construction of the indices, the raw scores of the items were added and were then averaged.

#### Students’ Perception of Teachers’ Wellbeing

This scale is an *ad hoc* measure constructed by the recommendation of expert judges who assessed the instruments used in this research. A brief scale was built on students’ perceptions of teachers’ wellbeing and welfare. This scale consists of five questions: “My teachers treat us well,” “My teachers like their work,” “My teachers are happy in this school,” “The teachers of this school treat each other well,” and “The teachers of this school have good working conditions.” The reliability analysis showed good performance, with a single factor and α = 0.82. The response range of the items ranges from 1 (“strongly disagree”) to 5 (“strongly agree”). For the construction of these indices, the raw scores of the items were added and then averaged.

#### Student Achievement and Performance

For the purposes of this research, achievement is understood as the probability of passing a class, as well as a general attendance to school. The main objective of this construct is to measure actual engagement with the school, reducing the probability of dropping out. A low achievement would lead to dropout (low probability of passing and low attendance). Performance is associated with the level of learning or success at school, and therefore is measured through a function of the grade point average (GPA). Low performance would lead to failing a class, or to mediocre educational outputs.

Student achievement was operationalized as the probability of course passing, estimated as (a) the student’s 5-year pass rate for the 2008–2012 period, and (b) student attendance, operationalized as the average student attendance in the 5-year period 2008–2012.

Student performance was defined as the grades obtained by a student in his school during the 5-year period 2008–2012.

Data for both variables was obtained from the school performance datasets of the Ministry of Education for the period 2008–2012 per each participant individual.

To create these variables, we merged the public datasets from the National Student Registry, with the database of primary sources collected in this study. The identity of the participants was recorded using the National Identity Number. Data linkage was carried out by Ministry of Education officers in charge of managing the National Student Registry. The Ministry uses a system called MRUN system^3^, which consists of an algorithm that hides the identity of individuals but allows them to be internally identified by creating a different MRUN for each national identity number, which does not alter over time.

Students’ performance was operationalized as a general academic score, measured through the yearly average marks obtained per student. The 5-year averages of this variable (2008–2012) were used to create a variable called “average grade estimation” using a linear model (OLS). This variable is defined for analytical purposes as “expected academic performance.” This procedure provided information for a total of 1,723 total cases, with 296 cases excluded.

#### Socio-Economic Measures: School Vulnerability Index

To measure SES of the school, we used the School Vulnerability Index (IVE by its Spanish initials). IVE is an index created by the Government of Chile to measure the degree of socio-economic vulnerability of students attending publicly funded schools, whether public or voucher. It is the official instrument for the assignation of school benefits across the country.

The IVE considers the children’s family’s socio-economic level, the educational level of the parents-tutors, the health condition of the student, the physical and emotional wellbeing of the student (using standardized tests) and the location of the school (urban or rural). On this basis, the IVE ranks schools according to how many of their students present a condition requiring special treatment and extra funding. IVE scores range from 0 to 100. Private schools are not included in the IVE scores; in these cases a value of 0 was assigned. IVE is updated yearly and classifies schools by the percentage of vulnerable children attended, which makes it a more sensitive measure than those based on averages.

Schools were classified as high (IVE < 10), mid (10 > IVE < 60) or low (60 < IVE) SES. These variables were coded as high = 1, mid = 2, and low = 3. All students inside a school share the same IVE.

Wang and Degol report that findings associated with SES at the school level are consistent, generally indicating that students attending schools with lower proportions of low SES children demonstrate not only higher levels of achievement but also greater growth in achievement over time (2006: 328). Therefore, a measure related to the proportion of vulnerable children attending the same school seems to be an adequate measure of the role of SES in achievement.

### Design and Analyses

#### Imputation of Missing Cases

To increase the number of cases for the variable “average grade estimation,” a model of imputation of missing cases was used through linear regression estimation (OLS) for the averages. The estimation protocol was based on the following conditions: (i) cases to be imputed cannot have more than two missing values; therefore, all cases with more than two missing values were not estimated; (ii) the imputation model is MCO with at least three adjacent values, and (iii) imputation of the central value was replaced by the average of the available values.

This procedure allowed the recovery of a total of 223 records, leaving 73 cases without value in the dependent variable. The total sample is 1,946 cases.

#### Data Analysis

Correlations, analysis of variance and structural equation models were used. Correlations were used to determine the degree of association that the different scales have among them. The analyses of variance allowed us to verify the existence of statistically significant differences between means of different groups. This analysis is relevant when using a stratified sample, which is composed of different groups, to determine whether there are significant differences in the results of the same variable.

SEM modeling consists of a hypothesis test to estimate the potential interrelationships between different constructs based on a theoretical model, as well as their indicators and measurement levels. The following indexes and values of goodness of fit were considered: Square Chi (Chi-Square), Comparative Fit Index (CFI) > 0.91; Root Mean Square Error of Approximation (RMSEA) < 0.05 (Byrne, 2009).

Analysis was done at individual level, with only SES measured at school level and used as a fixed variable for all students under the same school. All variables, except for SES are measured by student ratings.

Analysis was performed using SPSS Statistics 20 and SPSS Amos V.21.

## Results

### Descriptive and Correlational Analyses

The descriptive analysis shows few differences between the total and the matched sample. From here onward we will make us of the latter for the multivariate analysis (**Tables 2, 3**).

**Table 2:** Descriptive statistics full sample.

	*N*	Min.	Max.	Mean	Std. Dev.
Age	4620	14	27	15.6	0.78
Personal wellbeing index (averages)	4725	0.33	10	7.72	1.36
Brief Multidimensional students’ life satisfaction scale (averages)	4822	1	7	5.58	1.01
School social wellbeing (averages)	4597	1	5	3.53	0.61
School climate (averages)	4610	1	5	3.45	0.71
Perception of teachers’ wellbeing (averages)	4867	1	5	3.81	0.7
Positive and negative affect schedule-positive affects (averages)	4654	1	5	3.30	0.76
Positive and negative affect schedule-negative affects (averages)	4572	1	5	2.47	0.77
Positive and negative affect schedule-balance of affects (averages)	4472	-3	3.7	0.83	1.04

**Table 3:** Descriptive statistics matched sample.

	*N*	Min.	Max.	Mean	Std. Dev.
Age	1930	14	20	15.58	0.74
Probability of passing	1968	0.33	1	0.96	0.1
Attendance percentage	1968	44	100	92.80	5.06
Performance estimation	1946	3.86	7	5.69	0.588
Personal wellbeing index (averages)	1939	1.11	10	7.73	1.35
Brief multidimensional students’ life satisfaction scale (averages)	1972	1	7	5.55	1.02
School social wellbeing (averages)	1875	1.35	5	3.57	0.61
School climate (averages)	1895	1	5	3.53	0.69
Perception of teachers’ wellbeing (averages)	1997	1	5	3.88	0.67
Positive and negative affect schedule-positive affects (averages)	1920	1	5	3.35	0.74
Positive and negative affect schedule-negative affects (averages)	1890	1	5	2.54	0.77
Positive and negative affect schedule-balance of affects (averages)	1848	–3	3.6	0.81	1.06

All the values in the matched sample are in the expected range for the full sample, according to the minimum and maximum values. In **Table 3**, the probability of passing, attendance percentage, and performance estimation were included. The probability of passing is calculated on a percentage scale, with a mean of 0.96, showing high probabilities of passing. The same applies with attendance percentage (*M* = 92.80). Performance estimation was calculated according to the grade average of students in the Chilean scale (1–7), showing a mean of 5.69.

The correlational analysis shows that most variables show significant association. Nonetheless, school climate does not show a significant association with the performance estimation, nor with the probability of passing.

The strongest association is between PWI and BMSLSS. This is to be expected, considering that both are subjective wellbeing measures (**Table 4**).

**Table 4:** Correlations (*N* = 1968).

	1	2	3	4	5	6	7	8	9	10	11
(1) Performance estimation	–										
(2) Probability of passing	0.42^∗∗^	–									
(3) Attendance percentage	0.28^∗∗^	0.31^∗∗^	–								
(4) Personal Wellbeing Index	0.07^∗∗^	0.06^∗∗^	0.09^∗∗^	–							
(5) Brief Multidimensional students’ life satisfaction scale	0.05^∗^	0.05^∗^	0.08^∗∗^	0.68^∗∗^	–						
(6) School social wellbeing	0.14^∗∗^	0.09^∗∗^	0.09^∗∗^	0.40^∗∗^	0.46^∗∗^	–					
(7) School Climate	0.042	0.045	0.09^∗∗^	0.35^∗∗^	0.39^∗∗^	0.62^∗∗^	–				
(8) Perc. teachers’ wellbeing	0.09^∗∗^	0.09^∗∗^	0.13^∗∗^	0.29^∗∗^	0.32^∗∗^	0.49^∗∗^	0.67^∗∗^	–			
(9) Positive and negative affect schedule-positive affects	0.05^∗^	–0.009	0.039	0.38^∗∗^	0.37^∗∗^	0.25^∗∗^	0.22^∗∗^	0.18^∗∗^	–		
(10) Positive and negative affect schedule-negative affects	–0.06^∗∗^	–0.09^∗∗^	–0.038	–0.28^∗∗^	–0.32^∗∗^	–0.18^∗∗^	–0.10^∗∗^	–0.07^∗∗^	0.020	–	
(11) Positive and negative affect schedule-balance of affects	0.08^∗∗^	0.06^∗∗^	0.06^∗∗^	0.47^∗∗^	0.48^∗∗^	0.31^∗∗^	0.23^∗∗^	0.17^∗∗^	0.69^∗∗^	–0.71^∗∗^	–

*^∗^*p* < 0.05, ^∗∗^*p* < 0.01.*

### Analysis of Variance and Mediational Analyses

#### Academic Achievement

Testing the hypothesis about a relationship between subjective wellbeing and academic achievement began with the dichotomization in high and low subjective wellbeing (based on the mean of the BMSLSS), to assess the existence of significant differences between these groups. A mean difference test shows that students with high levels of subjective wellbeing attend (*t* = -2.395, *p* = 0.017) and pass (*t* = -2.749, *p* = 0.006) significantly more than students with low levels.

**FIGURE 1:**
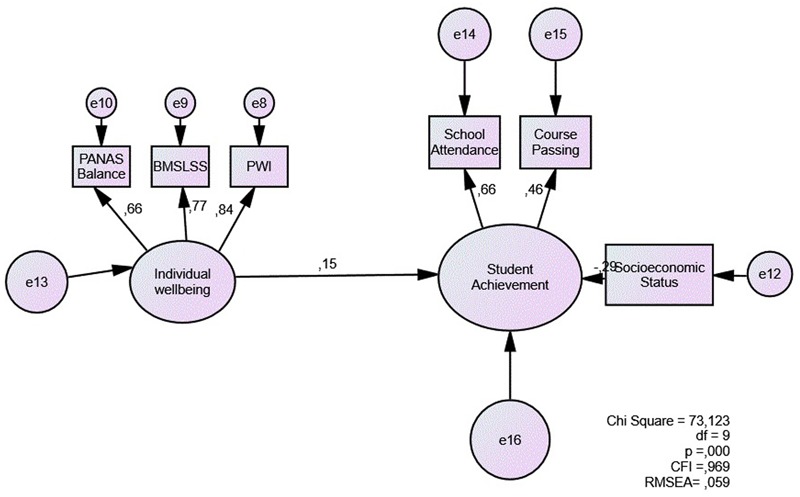
Direct model of subjective wellbeing as a predictor of student achievement.

For SEM analyses, the first step was to test subjective wellbeing as a predictor of academic achievement. We estimated a latent individual wellbeing variable expressed through the balance of affects (PANAS, PWI, and BMSLSS). This direct model shows that individual wellbeing predicts student achievement (0.15), controlling for the effect of SES. This model (**Figure 1**) shows a proper fit (RMSEA = 0.059, CFI = 0.969).

**FIGURE 2:**
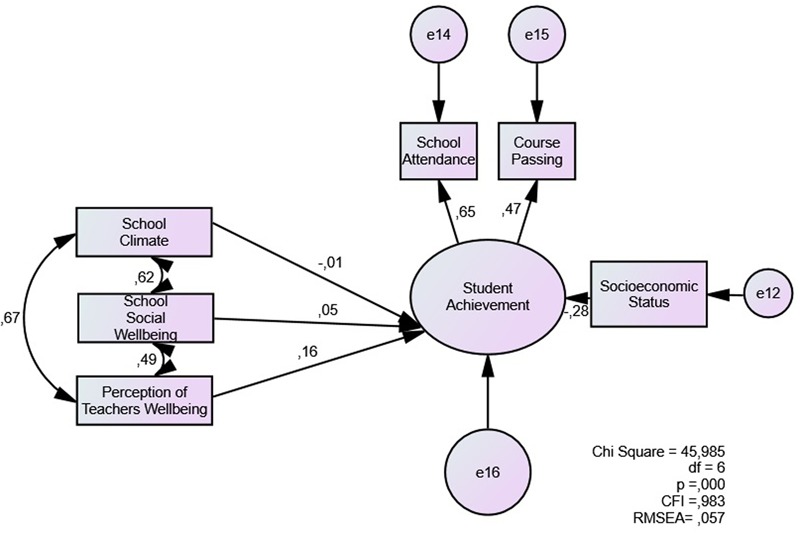
Direct model of school-related evaluations as predictors of student achievement.

Our second step was to assess the predicting values of school-related evaluations on student achievement. We tested these association in model 2 (**Figure 2**), which shows a proper fit (RMSEA = 0.057, CFI = 0.983). Perceptions of teachers’ wellbeing presents the higher coefficient (0,16), while school climate presents a negative association (–0,01). There are high correlations between school climate and the other two concepts.

**FIGURE 3:**
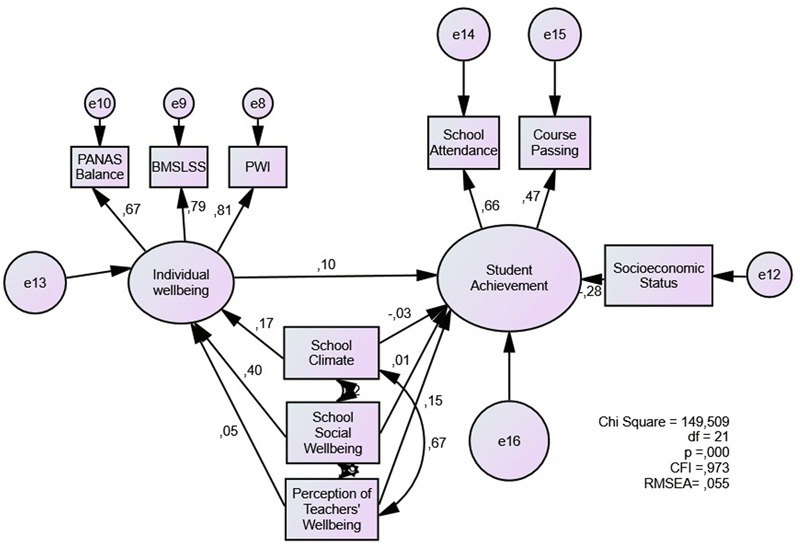
Individual subjective wellbeing as a mediator of the association between school-related evaluations and student achievement.

In Model 3 (**Figure 3**), we included individual subjective wellbeing as a mediating variable. The model shows an adequate fit (RMSEA = 0.055; CFI = 0.973). Individual wellbeing has a coefficient of (0.10), while the coefficients of the three school-related evaluations are reduced, with school social wellbeing showing the biggest decrease. Mediation significance was determined by using Sobel test (Sobel, 1982; MacKinnon et al., 2002), the results show significant mediation for both school climate and school social wellbeing. Teachers’ perception of wellbeing shows a direct association not mediated by individual wellbeing (**Table 5**).

**Table 5:** Mediation effects on achievement (*N* = 2019).

Variable	Direct effects			Indirect effect	*P*-value for mediation	
	β1 (SE)	β2 (SE)	β3 (SE)	β1 × β2 (SE)	*p = z1 × z2*	Association type
School social wellbeing	0,505 (0,037)^∗^	0,442 (0,186)^∗^	0,062 (0,245)	0,223 (0,095)^∗^	0,019	Mediated
School climate	0,189 (0,038)^∗^	0,442 (0,186)^∗^	–0,141 (0,235)	0,084 (0,039)^∗^	0,032	Mediated
Teacher’s wellbeing	0,056 (0,035)	0,442 (0,186)^∗^	0,757 (0,214)^∗^	0,025 (0,019)	0,184	Significant but
perception						disconnected association

*^∗^*p* < 0.05.*

#### Academic Performance

**FIGURE 4:**
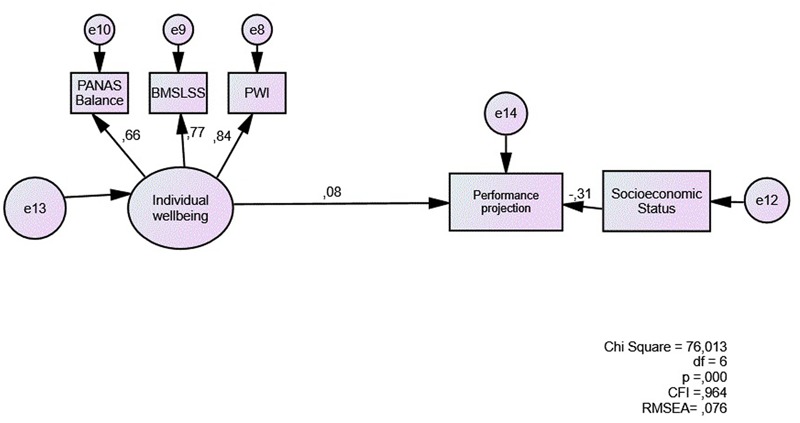
Direct model of subjective wellbeing as a predictor of student academic performance.

With academic performance as an outcome variable, the coefficient shown by individual wellbeing is lower than the one presented in Model 1 with student achievement (0.08) (**Figure 4**). Model fit is adequate (RMSEA = 0.076; CFI = 0.964).

**FIGURE 5:**
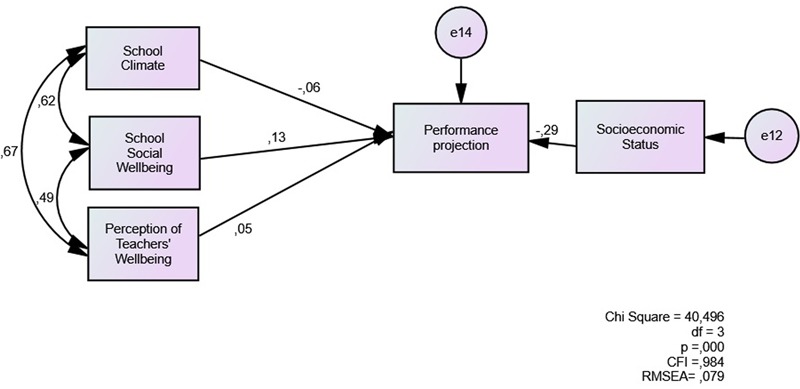
Direct model of school-related evaluations as predictors of student academic performance.

The next step was test the role of school-related evaluations on school performance (**Figure 5**). School social wellbeing shows the highest direct association with performance (0,13), while school climate shows a negative association. School climate shows high correlations with the other two predictors. This step improved the model fit (RMSEA = 0.079; CFI = 0.984).

**FIGURE 6:**
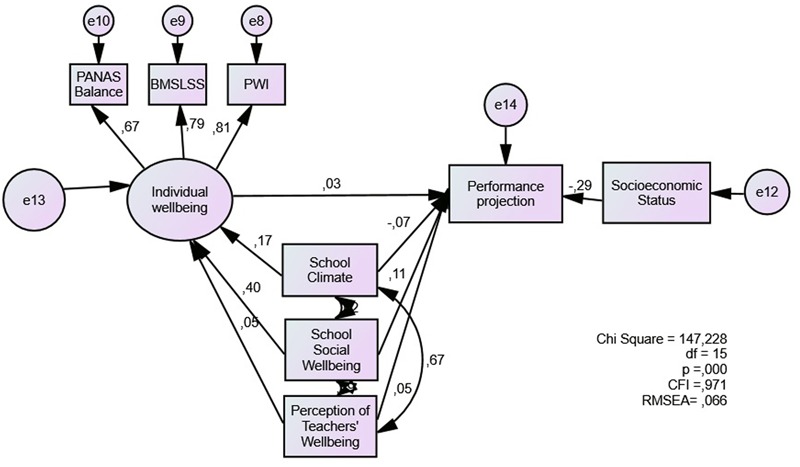
Individual subjective wellbeing as a mediator of the association between school-related evaluations and student academic performance.

Model 6 (**Figure 6**) presents the results for the full model of student performance, including individual wellbeing as a mediating variable. Individual wellbeing shows a small predicting value (0,03), while school social wellbeing has a higher direct association with performance (0.11). Model fit is acceptable (CFI = 0.971, RMSEA = 0.066). We found no mediating role of individual wellbeing for the prediction of student performance (**Table 6**).

**Table 6:** Mediation effects on performance (*N* = 2019).

Variable	Direct effects			Indirect effect	*P*-value for mediation	
	β1 (SE)	β2 (SE)	β3 (SE)	β1 × β2 (SE)	*p = z1 × z2*	Association type
School social wellbeing	0,505 (0,037)^∗^	0,025 (0,023)	0,106 (0,03)^∗^	0,013 (0,012)	0,279	Direct
School climate	0,189 (0,038)^∗^	0,025 (0,023)	–0,055 (0,029)	0,005 (0,004)	0,288	No association
Teacher’s wellbeing perception	0,056 (0,035)	0,025 (0,023)	0,04 (0,026)	0,001 (0,002)	0,369	No association

*^∗^*p* < 0.05.*

The results show that individual wellbeing has a direct effect on school performance: when subjective wellbeing increases a standard deviation, achievement measured in attendance and approval of the school year contributes 0.10 standard deviation. This relation is obtained by controlling the already known effect of SES of schools. On the other hand, when subjective wellbeing increases by one standard deviation, academic performance rises by 0.08 standard deviation. It also mediates the relationship between school social wellbeing and school climate with students’ achievement. In the full model specification, perception of teachers’ wellbeing presents a direct association with achievement, while school social wellbeing does the same with performance.

## Discussion

This study aimed to determine the relationship between high school students’ individual wellbeing, school-related evaluations, academic achievement, and school performance. Findings support our initial hypotheses of positive associations between wellbeing measures and academic achievement and performance. Findings provide partial support for the mediating hypothesis of individual wellbeing on school-related evaluations. We found evidence for a partial mediation of individual wellbeing on the association between school social wellbeing and school climate with the outcome variables of school achievement.

Models reveal that the multidimensional scales of individual wellbeing allow the identification of effects of subjective wellbeing on academic achievement and student performance. The PWI and BMSLSS scales are theoretically complementary and increase the fit of the model for the determination of subjective wellbeing. Together with PANAS, they provide a global vision of individual subjective wellbeing.

The models also highlight the predicting role of evaluations made by children about the school on academic achievement and performance. These results support the results previously reported in the literature, although it is important to highlight the high correlation between the concepts school social wellbeing and school climate. This high association is a relevant point considering that school climate is a still a broad conceptual umbrella for several processes occurring in schools, whereas school social wellbeing represents a defined and clear concept rooted in the eudemonic tradition of wellbeing.

Individual wellbeing affects the relationship between school social wellbeing and school climate with the outcome variables of school achievement, meaning that both evaluations of school culture are mediated by the life outlook of adolescents. These variables are socio-affective variables embedded in the school context (Benbenishty and Astor, 2005; Espelage and Swearer, 2010), but are not regularly analyzed from the perspective of children and their general concerns. According to Casas et al. (2013), there is no other more direct and valid method to assess children’s perspectives than turning directly to them. Children are key informants of their lives and relevant agents in providing data on the realities they experience. As we can see from our results, their individual wellbeing affects the way they experience school and how this experience translates into achievement.

Of course, our findings also highlight the role of school-related evaluations which are not related to individual wellbeing: perceptions of teachers’ wellbeing have a direct association with achievement, meaning that the dyadic relationship between teacher and student can have a greater influence than individual general concerns for student achievement. Regarding the estimation of academic performance, school social wellbeing is the best predictor, not being mediated by individual wellbeing.

Finally, SES is a relevant variable for the prediction of educational achievement and performance, confirming what has already been widely described in the Latin American literature (UNESCO, 2008, 2016; Valenzuela et al., 2014).

We have tested hypotheses regarding the direct effect of individual wellbeing on academic achievement and its mediating role on school-related evaluations. These results allow us to say with high confidence that individual wellbeing is a component that increases the probability of attending school and passing grades, as well as obtaining higher marks. Therefore, it is a protective factor, reducing the probability of dropout and school delay in adolescents.

However, further analyses of this relationship are needed to understand which underlying mechanisms, at the individual and collective level, would explain this influence on achievement and performance. We propose that improvement can be fomented through the promotion of positive mental health of the school community, without forgetting the role of individuals. Improving schools involves a healthy school environment with a high perception of school social wellbeing, fostering and supporting teacher-student relationships as well as boosting both students’ and teachers’ individual wellbeing (Wubbels, 2011; Wubbels et al., 2015).

## Ethics Statement

This study was carried out in accordance with the recommendations of the National Commission of Science and Technology of Chile with written informed consent from all subjects. All subjects gave written informed consent in accordance with the Declaration of Helsinki. The protocol was approved by the ethics committee of Pontificia Universidad Católica de Valparaíso.

## Author Contributions

VL co-lead the research, drafted the first version of the manuscript and reviewed the final one. JO co-lead the research, ran the analyses and drafted the second version of the manuscript. MB directed the original research project, conducted a literature review, and commented on an earlier version of the manuscript. JT improved the literature review. DO and MM provided research assistance. PA and CC commented on an earlier version of the manuscript.

## Conflict of Interest Statement

The authors declare that the research was conducted in the absence of any commercial or financial relationships that could be construed as a potential conflict of interest.

## Footnotes

1 One of the policies that has contributed to this phenomenon is the promulgation in 2003 of the Law N°19.876, which establishes secondary education as compulsory and free in public schools.

2 Estimation based on the enrolment of the classrooms surveyed.

3 Mask of the Unique Birth Registry number, *Rol Único de Nacimiento*, in Spanish.
